# The unfolded protein response in virus infections

**DOI:** 10.3389/fmicb.2014.00518

**Published:** 2014-09-30

**Authors:** Shiu-Wan Chan

**Affiliations:** Faculty of Life Sciences, The University of ManchesterManchester, UK

**Keywords:** unfolded protein response, endoplasmic reticulum stress, ERAD, autophagy, innate immunity, gene therapy, pathogenesis, virus-host interaction

Unfolded protein response (UPR) is a cellular homeostatic response to endoplasmic reticulum (ER) stress. Increasing evidence suggests an intimate relationship between virus and UPR. This research topic collated a number of review articles and original research article, in an attempt to highlight how viruses interact with the host UPR in the establishment of acute, chronic and latent infections.

Virus infection represents an arm race between virus and the host. On one hand, the host mobilizes the UPR in an attempt to restrict virus infection. On the other hand, virus subverts or even manipulates the UPR to assist in its own infection. The consequence of this is that the UPR is often skewed during virus infections to either favor virus elimination or virus invasion. Whoever won, the outcome could be pathogenic. The relationship between virus and UPR and its associated autophagy is being addressed in three reviews focusing on RNA viruses, as their life cycles are closely associated with the ER (Blazquez et al., 2014; Fung and Liu, 2014; Jheng et al., 2014). Miguel Martin-Acebes and his group focuses on flaviviruses whereas To S. Fung and Ding X. Liu focus on coronaviruses. Jim-Tong Horng's group takes a closer look at virus interaction with autophagy and also discusses the potential of targeting UPR and autophagy as novel anti-virals.

In contrast to acute virus, one can only imagine that virus establishing a life-long chronic infection may interact with the host UPR in a completely different way to maintain an environment favorable for virus survival. Two reviews presented by Shiu-Wan Chan and Norica Branza-Nichita's group on hepatitis C virus and hepatitis B virus, respectively, shed light on how persistent virus interacts with the host UPR to benefit establishment of a chronic infection and how chronic activation of the UPR leads to diseases (Chan, 2014; Lazar et al., 2014).

UPR is prevalent in viruses establishing latent infections such as herpesviruses. Herpesvirus is an ancient virus. During its course of millions of years of co-inhabitation with its host, herpesvirus has borrowed a number of molecules from its host to be used in its life cycle. There is no exception in UPR, in which herpesviruses also share molecular mimicry with the UPR molecules and utilize UPR to set up lytic infection and to break dormancy, suggesting that interaction of virus with host UPR may be very ancient. Varicella-zoster virus (VZV) possesses the smallest genome of human herpesviruses and lacks some genes used by other herpesviruses to manipulate the UPR. The key question is therefore whether VZV UPR induction is merely a host response or a result of viral manipulation. By using a UPR PCR array, John Carpenter and Charles Grose demonstrated VZV differentially induced the UPR to expand the ER to cope with viral glycoprotein synthesis (Carpenter and Grose, 2014). This study also uncovered VZV upregulation of an unusual UPR molecule, the cAMP responsive element binding protein H. Clearly, this will pave the way to future studies to disclose the relationship between VZV and UPR.

ER-associated degradation (ERAD) is part of an UPR functioning to extract unfolded/misfolded proteins from the ER into the cytosol for proteasomal degradation. Not surprisingly, this process is also targeted by virus. Jaquelin Dudley and her group re-captures the ERAD process in details followed by an illustration of how viruses exploit this process (Byun et al., 2014). First, viruses can simply mobilize the ERAD to degrade important immune molecules or viral envelope glycoproteins to evade innate and adaptive immune responses. At a more intimate level, some viruses have actually incorporated ERAD into their life cycles for viral protein and even virion maturation. It is fascinating how naked polyomaviruses will make a *de tour* to the ER for ERAD-assisted uncoating before re-entering the cytosol *en route* to the nucleus. Lastly, viruses can interfere with ERAD tuning and hijack certain ERAD cargo into forming double membrane vesicles as sites of virus replication.

UPR has emerged to be more than a homeostatic cellular response to virus infections. UPR has been intimately linked to innate immunity; whether by modulating innate immunity or as part of the innate immunity. Innate immunity is initiated by the sensing of “danger signals” by host pattern recognition receptors (PRRs), culminating in the release of interferon, which in turn activates the professional virus killer, one of which is RNase L. One of the proximal UPR sensors, inositol-requiring enzyme 1 (IRE1), is evolutionarily related to RNase L. In the review of Sankar Bhattacharyya, he provides a structural and functional comparison between IRE1 and RNase L and comments on a potential anti-viral function of IRE1 by the creation of “danger signals” via the regulated IRE1-dependent decay (RIDD) pathway (Bhattacharyya, 2014). An important question remains as to whether UPR represents a new tool for sensing viruses or select UPR molecules are merely being co-opted in “microbial stress response.” This is being addressed in Judith Smith's review, in which she provides a critique on the intersection of the UPR with the inflammatory pathways and innate immunity and offers an insight into UPR-PRR synergy as an evolutionary adaptation to ensure specificity of anti-viral responses (Smith, 2014).

It is increasingly popular to use viruses in clinical applications such as gene therapy and oncolytic virotherapy. The use of viral vectors/viruses in the clinics will not be valid without a thorough understanding of virus-host interaction. Giridhara Jayandharan and his group presents a review on the emerging impact of UPR on gene therapy and how the understanding of this will allow us to exploit and improve the use of viral vectors in gene therapy (Sen et al., 2014).

To date we are still at the sprouting stage of understanding this virus-host interaction. We hope that this selection of articles will provide a foundation to spark more interest in this research area. This will not only lead to a deeper understanding of virus infection and pathogenesis but will also unravel novel anti-viral mechanisms. Eventually it will help to unlock novel anti-viral targets and may also impact on optimizing the use of viruses in the clinics.

## Conflict of interest statement

The author declares that the research was conducted in the absence of any commercial or financial relationships that could be construed as a potential conflict of interest.
